# The tail dependence of the carbon markets: The implication of portfolio management

**DOI:** 10.1371/journal.pone.0238033

**Published:** 2020-08-28

**Authors:** Fang Zhang, Zhengjun Zhang

**Affiliations:** 1 Capital University of Economics and Business, Beijing, China; 2 University of Wisconsin, Madison, Wisconsin, United States of America; Shandong University of Science and Technology, CHINA

## Abstract

Emission trading scheme (ETS), the most popular market-based instrument, is widely used to solve carbon emission problems in the world. With the development of carbon market, carbon asset has been a popular financial product to invest and the risk management becomes important for government, regulated enterprises and other investors. As carbon prices have tail characteristics, this paper explores the extremal risks between carbon markets in US, Europe and China using tail dependence correlation coefficients. The empirical analysis demonstrates the tail dependence structure between carbon markets in US. and Europe is the same sign, which indicates that it is unwise to hold these two carbon assets as a portfolio. Moreover, the co-movements between European Union Emission Trade Scheme (EU ETS) and China’s carbon markets are partly significant, and the operation mechanisms in China should be improved. In addition, the tail dependence test among the carbon pilots in China shows diversity. Hubei carbon trading pilot, located in central China, has extremal dependence with all other selected pilots for its regulatory program operation. The findings give insight to the carbon market regulars to improve the operation mechanism and are also useful for the investors to manage their portfolios, policymakers to make practically applicable regulations, and relevant organizations to develop procedures.

## 1. Introduction

Climate change has deep influence on environmental issues, the economy and human health in the world. Green House Gas (GHG) emissions are the main source of climate change and global warming [1]. Facing the situation, many countries and regions implement the Emission Trading Scheme (ETS) to achieve emission mitigation goals set by the Kyoto Protocol, Copenhagen Accord and Paris agreement. ETS is an effective instrument to control GHG emissions [2]. A number of countries and regions employ ETS to control emissions. Carbon markets have been rapidly developed and steadily expanded in recent years. California Cap and Trade Program (CA CAT) is the economy-wide carbon trading scheme [3]. Under the Assembly Bill 32, it sets a statewide limit on sources responsible for 85% of California’s GHG emissions. EU ETS is the oldest carbon market in the world, establishing in 2005. The EU ETS has experienced its third phases since its build [4]. China has built seven carbon trading pilots, aiming at accumulating operation experience for national carbon market establishment. Although the carbon markets are emerging markets, they have become an important part of the global financial market. Learning the price mechanism of the carbon markets helps the regulated enterprises allocate assets and adjust their production strategies, which influences the emission mitigation effectives.

For carbon market studies, one strand of the existing researches mainly focuses on carbon price determinants in the European Union Emissions Trading System (EU ETS). Carbon prices in the EU ETS are affected by multiple variables, such as energy market, financial markets, extreme weather, policy measures, regulation scheme and so on [5–14]. Reboredo [15] employed a multivariate conditional autoregressive range model to find and examine the dynamic relationship between the EU ETS and oil markets. The results showed that the equilibrium relationship is different for the two phases of the EU ETS [16]. Aatola [17] used several econometric models to discover the relationship between carbon prices and energy prices, such as gas prices, electricity prices and coal prices. When emissions occurred seriously, CO_2_ price is more sensitive to the gas price [18]. Macroeconomy is also an important factor for carbon price volatility [6,19]. Chevallier [20] used a Markov-Switching VAR model to investigate the link between macroeconomy and carbon prices. Carbon futures has a remote correlation with macroeconomic variables, such as stock market and bond [21]. Except the above factors, extremal weather and special events could also affect carbon prices. For instance, the leakage of carbon emission quota of the Czech Republic, France, and Sweden in 2006 led carbon prices dropped sharply.

Another strand of researches is about the correlation between carbon market and other markets. The present literatures can be separated into two categories. The first is the correlation between carbon market and energy markets. DCC-TGARCH model, full BEKK-GARCH model and MS-DCC-GARCH are applied to estimate the dynamic volatility spillover between carbon prices and fossil energy prices [22,23]. Different copula models were used to find the dependence between the EUA price and crude oil price during the second phase in the EU ETS. It was found that the carbon market attracts investors to reduce downside risk in oil markets [24]. Ji et al [25] adopted a systemic time series method to explore the information linkages and spillover between carbon market and energy market. Different markets show significant and asymmetric tail dependence with carbon market [26]. The second category is learning the relationship between carbon market and financial market [27]. For instance, carbon prices drop leads to the negative impact on stock returns of carbon-intensive industries [28]. Polluting sectors returns respond to the carbon prices diversely for different industries [29,30]. Not all firms would benefit from carbon markets in the short term, but it is possible to be profitable in the long term [31]. The co-movement and asymmetric dependence relationship between China’s carbon emission allowances and gasoline market are investigated [32,33]. Uddin et al., [34] used c-vine conditional VaR and c-vine copula to analyze the dependence structure and spillover effects across China’s carbon market and energy markets. Zhang et al., [35] measure the dependence between EU ETS and international financial markets.

As to the operation mechanism, a few studies analyze the comparison of the existing carbon markets. Shen et al. [36] looked back to the CA CAT and drew insights for China’s carbon markets involving legal basis, institutional arrangement and allowances distribution. Existing studies also concentrate on single carbon market risk and market efficiency [37]. Comparing with clean development mechanisms (CDM), the EU ETS has a higher risk [38]. Extreme value theory (EVT) and Value at Risk (VaR) are commonly used in carbon market risk analysis. It is found that risk of the EU ETS is higher in the first phase than in the second phase, the downside risk is higher than the upside risk for the carbon market [39]. Carbon markets have been proved to be an alternative investment way to make profits and disperse asset risks [4,22,40]. The significance of carbon price risk management becomes significant with the development of carbon market. Fan [41] calculated hedge ratios and investigated the hedging effectiveness in the EU ETS. A time series analysis demonstrated that most speculative activities takes place in the front contract, however, the hedging demand concentrated in the second-to-deliver futures contract [42].

Reviewing the available literatures, we find that the previous studies mainly focus on carbon price analysis. Regarding to the risk management analysis, the existing studies concern single carbon market risk analysis, and ignore studying the dependence between carbon markets. However, the extreme cases would cause great volatility in carbon markets and carbon prices have the tail distribution characteristics. For instance, extremal energy prices fluctuation and special events could affect carbon prices [24,39]. Thus, learning the extremal risks of carbon markets is essential for asset allocations, investment strategies, and risk management. Modelling tail dependence of different carbon markets help investors understand the relationship between carbon markets and make risk assessment. Firstly, studying the tail dependence between carbon markets and grasping change law between carbon prices help investors choose investment portfolios reasonably, diversify financial risks and formulate effective policies. Secondly, carbon prices can be considered as part of production cost for enterprises. Thus, learning the dependence structure of carbon markets could help enterprises identify carbon price risk and adjust their production strategy in the long run. Thirdly, finding out the reasons for asymmetric tail dependence between carbon markets is conductive to studying the carbon markets and find out the problems of the involved carbon markets, which helps policy makers adjust management and operation instructions.

This paper aims at exploring the tail dependence structure between carbon markets and giving a clear vision of carbon markets using a novel method, tail quotient correlation coefficient (TQCC). TQCC is a method to identify whether there exists extremal dependence between the selected two assets. Generally, this study contributes to the existing literature in the following aspects. Firstly, this paper explores the extremal co-movement between carbon markets in China. Learning the dependence of different carbon markets helps investors allocate carbon assets reasonably. Secondly, we examine and compare the correlation between EU ETS and China’s carbon markets to shed light on the operation problem of China’s carbon markets. Thirdly, learning the carbon market risk is beneficial to the management of carbon market and regulated industries. Understanding carbon markets dependence structure is conducive to improving carbon market efficiency, which contributes to achieving low-carbon development and energy saving for the covered enterprises. This paper finds out the problems and made implication to the policy makers and market participants.

This paper is unfolded as follows. Section 2 introduces the methodology; Section 3 is the data selection; Section 4 discusses the results and findings; Section 5 gives the conclusion and implication.

## 2. Methodology

The analysis of cross-market linkages needs knowledge of the dependency of the selected markets. EVT is an efficient approach to analyze extremal issues. Financial assets are demonstrated to appear tail dependence. In this paper, we apply extreme value theory to calculate the tail dependence between carbon markets.

The idea of tail independence between two random variables with identical marginal distributions was initially introduced by Sibuya [43]. The basic notion of tail dependence is that the probability of a two-dimensional random vector occurs extremal values at the same time. Zhang et al. [44] states that the identically distributed random variable pair (X, Y) is tail dependence if

$$\lambda =\underset{u\to x_{F}}{\text{lim}}P(Z>u|T>u)=0$$

where T_F_ = sup{t ∈ R: P(T ≤ t) < 1}. λ is called the bivariate upper tail dependence index which quantifies the degree of dependence of the bivariate tails. There is extremal co-movement between T and Z if λ > 0.

As to its extension, tail dependence estimation for the multivariate case was developed [45], joint tail models was induced for near independence pair when the threshold was fixed [46]. Regarding the test statistic of tail dependence, the research based on the null hypothesis of tail dependence is developed [47]. Contrary to the former hypothesis, the tail quotient correlation and the tail independence test statistics based on the null hypothesis of tail independence were generated [48]. Tail quotient correlation coefficient (TQCC) was first proposed to measure tail dependence between two random variables using random thresholds which can diverge to infinity [44]. A practically workable threshold selection was also suggested in the paper. TQCC is a method to study tail dependence between two time series sequences. This method is based on extreme value theory, which is applicable to the data with tail distribution characteristic. In fact, carbon prices have the tail distribution characteristics. In this paper, we will employ TQCC to study the tail dependence structures of carbon markets in the world.

The main steps for estimating TQCC are as follows: The first step is to fit the autoregressive model (AR) and the Generalized Autoregressive Conditional Heteroscedasticity model (GARCH) to the time series to filter volatilities. GARCH(q,p) can be specified as:

$$y_{t}=\rho_{0}+\rho y_{t-1}+\varepsilon_{t}$$ (1)

$$\varepsilon_{t}=\mu_{t}\sqrt{h_{t}}$$ (2)

$$h_{t}=\alpha_{0}+\alpha_{1}\varepsilon_{t-1}^{2}+\alpha_{2}\varepsilon_{t-2}^{2}+\cdots +\alpha_{q}\varepsilon_{t-q}^{2}+\beta_{1}h_{t-1}+\beta_{2}h_{t-2}+\cdots +\beta_{p}h_{t-p}$$ (3)

where α_0_ is a constant term, α_q_ is the coefficient of the q-order autoregressive conditional heteroscedasticity model (ARCH) term, and β_p_ is the coefficient of the p-order GARCH term. After that the adjusted index time series (μ_t_) would be stationary and could be applied the subsequent EVT estimation. The adjusted time series are also called pseudo-observations.

The second step is fitting the exceedances into extreme value distribution. The exceedances are defined as the observations over a threshold. The exceedances series then is fitted into an extreme value distribution. Generalized Pareto Distribution (GPD) and Generalized Extreme Value distribution (GEV) are common extreme value distribution to fit exceedances. GEV, representing three extreme value types including Gumbel, Fréchet, and Weibull, is more general than GPD. GEV and a point process approach are employed in this study, and the GEV formula is as follows:

$$H(x)=\text{exp}({-(1+\xi \frac{\text{x}-\mu }{\psi })}_{+}^{-1/\xi })$$ (4)

where μ is the location parameter, ψ > 0 is the scale parameter, and ξ is the shape parameter.

The third process is transforming the pseudo-observations series to unit Fréchet scale with the three estimated parameter extracted from the GEV fitting. The unit Fréchet scale can be obtained using the transformation -1/log{H(x)} applying to those observations having exceeded the chosen thresholds.

After the transforming step, we could explore whether tail dependence structure exists between the selected series. Next, we introduce the TQCC formula.

The TQCC could be estimated by the following formula [49]:

$$q_{u_{n}}=\frac{{max}_{1\leq i\leq n}\{max(X_{i},u_{n})/(Y_{i},u_{n})\}+{max}_{1\leq i\leq n}\{max(Y_{i},u_{n})/(X_{i},u_{n})\}-2}{{max}_{1\leq i\leq n}\{max(X_{i},u_{n})/(Y_{i},u_{n})\}\times {max}_{1\leq i\leq n}\{max(Y_{i},u_{n})/(X_{i},u_{n})\}-1}$$ (5)

where ${\left\{\left(X_{i},Y_{i}\right)\right\}}_{i=1}^{n}$ is a random sample of unit Fréchet random variables (X, Y). The value of $q_{u_{n}}$ is the probability that one carbon market experiences a big price decrease (negative return over u) given another carbon market already suffered a big price decrease (negative return over u). For properties of $q_{u_{n}}$ and its good performance in testing and measuring tail dependence, we refer readers to [44].

## 3. Data selection

In this study, we select data from CA CAT, EU ETS and China’s ETS pilots. Beijing ETS, Shanghai ETS, Shenzhen ETS, Guangdong ETS and Hubei ETS are selected as representatives for China’s carbon markets. Tianjin and Chongqing carbon market are excepted from this paper since their inactive trading. Table 1 shows the comparison of the selected carbon markets.

**Table 1:** Comparison of China’s ETS, EU ETS and CA CAT.

Carbon market	Emission cap	Allowance distribution	Dynamic management
CA CAT	488 MtCO_2_(the first phase)	Benchmarking approach	yes
	1147 MtCO_2_(the second phase)		
	1039(the third phase)		
EU ETS	2181 MtCO_2_(the first phase)	Grandfathering approach (the first phase) Auction approach (the third phase)	yes
	2083 MtCO_2_(the second phase)		
	1720(the third phase)		
Beijing	50 MtCO_2_(in 2013)	Mostly free distribution method	ex-post dynamic adjustment
	50 MtCO_2_(in 2015)		
Shanghai	160 MtCO_2_(in 2013)	Mostly free distribution method	ex-post dynamic adjustment
	160 MtCO_2_(in 2015)		
Shenzhen	33 MtCO_2_(in 2013)	Mostly free distribution method	ex-post dynamic adjustment
	33 MtCO_2_(in 2015)		
Guangdong	388 MtCO^2^(in 2013)	Mostly free distribution method	ex-post dynamic adjustment
	408 MtCO_2_(in 2015)		
Hubei	324 MtCO_2_(in 2013)	Mostly free distribution method	ex-post dynamic adjustment
	281 MtCO_2_(in 2015)		

We select price of CCU (California carbon unit, the trading good in CA CAT), price of EUA (Emission unit allowance, the trading good in the EU ETS), and carbon prices in five carbon trading pilots in China (Shenzhen, Hubei, Beijing, Shanghai and Guangzhou). The data is of daily frequency and retrieved from Wind database. The data pairs are displayed as follows:

- For the CA CAT and the EU ETS, the time horizon for these two indices is from January 3^rd^ 2012 to December 22^nd^ 2017, including 1482 valid observations.
- For the EU ETS and China’s carbon trading pilots, the specific data period selections are shown in Table 2.
- The pairs of China’s carbon trading pilots. The data selections are reported in Table 3.

**Table 2:** Data period selection for the EU ETS and China’s carbon trading pilots.

Pair	From	To	Valid Trading Days
(EUA, SZEA)	2013/8/5	2017/12/29	986
(EUA, HBEA)	2014/4/28	2017/12/29	876
(EUA, BJEA)	2013/11/29	2017/12/29	640
(EUA, SHEA)	2013/12/3	2017/12/29	607
(EUA, GDEA)	2013/12/20	2017/12/29	718

**Table 3:** Data period selection among China’s carbon trading pilots.

Pair	From	To	Valid Trading Days
(SZEA,HBEA)	4/28/2014	2017/12/29	817
(SZEA,BJEA)	11/29/2013	2017/12/29	612
(SZEA,SHEA)	12/3/2013	2017/12/29	590
(SZEA,GDEA)	12/20/2013	2017/12/29	677
(HBEA,BJEA)	4/28/2014	2017/12/29	558
(HBEA,SHEA)	4/28/2014	2017/12/29	516
(HBEA,GDEA)	5/5/2014	2017/12/29	681
(BJEA,SHEA)	12/5/2013	6/29/2016	437
(BJEA,GDEA)	3/11/2014	8/31/2016	480
(SHEA,GDEA)	3/11/2014	6/29/2016	445

Beijing emission allowance is denoted by (BJEA), Guangzhou emission allowance by (GZEA), similarly, Hubei emission allowance by (HBEA), Shanghai emission allowance by (SHEA), Shenzhen emission allowance by (SZEA).

## 4. Results and discussion

### 4.1 Data processing

#### Fit GARCH

To begin with the empirical analysis, the carbon price time series are firstly converted to the logarithmic return series. Financial time series has a certain degree of autocorrelation and heteroscedasticity, while the EVT requires the stationary series. Thus we made return series filtered by GARCH model. After fitting GARCH, we can filter out the volatility and get the stationary time series. Moreover, the conditional standard deviations are extracted from the return series. The return series is divided by conditional standard deviations, and then we obtain the pseudo-observations.

#### Fit GEV

Fitting exceedance data with the pseudo-observation series over a threshold (u) to a GEV distribution. We choose 1.2 as the threshold, which leaves about ten percent, which has been used in the literature for intermediate sample sized data, of the pseudo-observation above or below the threshold. In order to explore tail dependences in the bull and bust markets, we fit GEV using both negative and positive pseudo-observation series separately.

#### Transform to Fréchet scale

To separate dependency from marginal distributional features, the pseudo-observation series are transformed to Fréchet scale using the three parameters obtained from the above GEV step.

#### Tail dependence test

After transforming to the unit Fréchet scale, the series could be employed the tail dependence test to find whether there is a tail dependence structure between the paired series. The test significance level is 0.05, and the empirical results are summarized in section 4.2.

### 4.2 Tail dependence results and discussion

The estimations of tail dependence tests are reported as follows. The constituent time series for the exceedance pair include the same sign (i.e., n*n, means negative & negative or p*p, means positive & positive). The pairs also include the opposite sign pair (i.e., n*p, means negative& positive or p*n, means positive& negative).

#### 4.2.1 Tail dependence results of CA CAT and EU ETS

**Fig 1:**
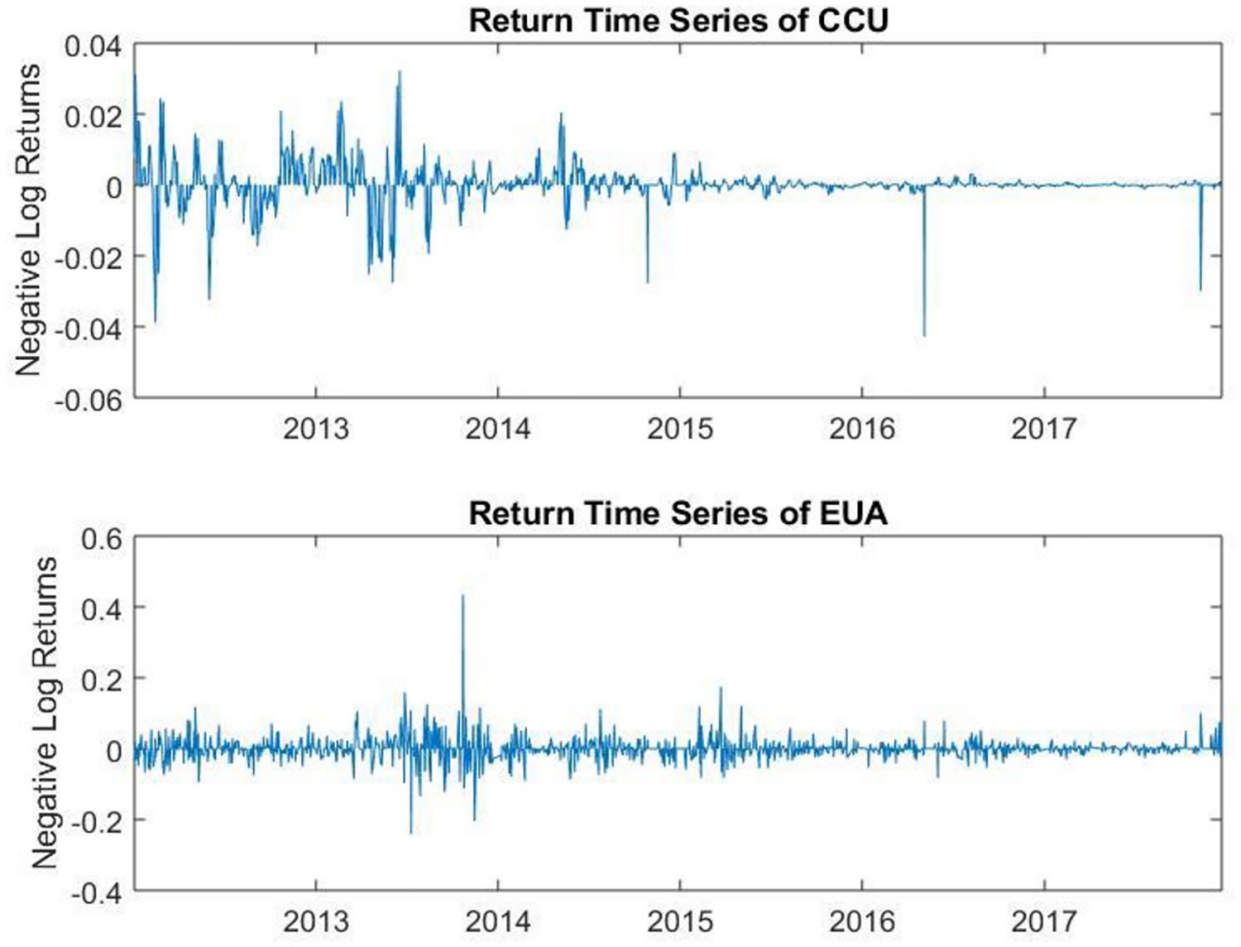
Negative logarithmic return time series of the two series: CCU and EUA.

Following the tail dependence modeling and testing process mentioned in Section 4.1. The negative logarithmic returns of the CCU and EUA are plotted in Fig 1. From the figure, we could find out the extremal return, jumps in returns and clustering in volatility of the two series.

**Fig 2:**
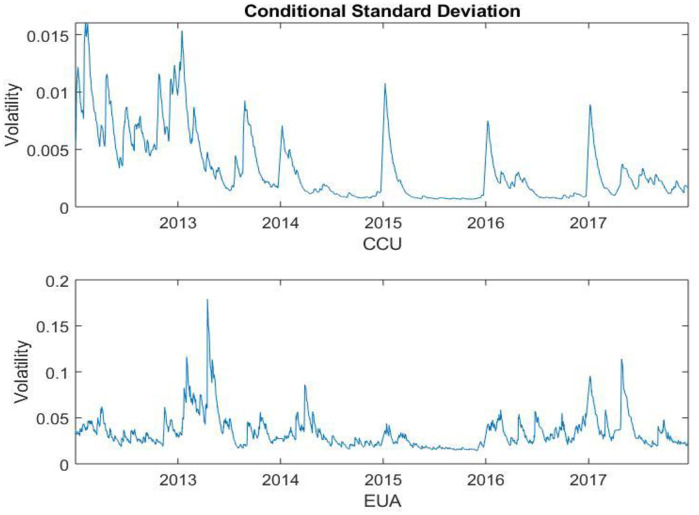
The conditional standard deviation time series using GARCH(1,1) filtering process.

As indicated, the EVT modeling requires the sequences to be stationary. Thus, we proceed the GARCH (1,1) model to filter the series. The GARCH filtered the conditional standard deviation of the two series are presented in Fig 2. After GARCH process, the return series show fluctuation in volatility at certain degree. The results indicate that the two indexes experience some familiar jumps.

The next step is to fit the exceedance data to GEV model. The obtained three estimated parameters are listed in Table 4. The shape parameter ξ is larger than zero. The confidence intervals for the ξ are positive, and do not include zero. This can be treated as strong evidence that the probability distributions of the carbon asset returns are heavy tail.

**Table 4:** The estimated parameters obtained from GEV.

	Nu	μ	ψ	ξ
CCUn	226	8.0279	0.8961	0.157
CCUp	187	10.7377	1.5465	0.3425
EUAn	154	5.7817	0.8536	0.35
EUAp	155	4.9229	0.458	0.2156

1: CCUn means negative return of CCU; EUAn means negative return of EUA

2: CCUp means positive return of CCU; EUAp means positive return of EUA

After the GEV fitting, we transform the pseudo-observation series to Fréchet scale and estimate the tail index using Eq (5). Table 5 summarizes tail dependence tests results for CCU return series and EUA return series. Different direction pairs are indicated from the third column to the sixth column. For instance, for the third column, (CCU_n_, EUA_n_) indicates the quotient correlation of the negative exceedance series of the CCU and the negative exceedance series of EUA. (CCUn, EUAp), in the fourth column, is the quotient correlation of the negative exceedance series of the CCU and the positive exceedance series of EUA.

**Table 5:** Tail dependence test result for CCX and EU ETS.

	n*n	n*p	p*p	p*n
Current day	0.1591*	0.0458	0.1999*	0.0094
	(0.0004)	(0.2556)	(0.0000)	(0.8542)
1-day lag	0.1297*	0.0503	0.1115*	0.0060
	(0.0006)	(0.1878)	(0.0062)	(0.9244)
2-day lag	0.1460*	0.0483	0.1059*	0.0431
	(0.0005)	(0.1787)	(0.0072)	(0.1996)
5-day lag	0.1461*	0.0326	0.0926*	0.0050
	(0.0007)	(0.2953)	(0.0062)	(0.9394)

1: n means negative return; p means positive return

2: PV represents P value.

3: * means significant at 0.05.

4: Lag trading day is for California carbon market.

The results indicate that the same-sign-pairs of CCU and EUA have the extremal co-movement. CA CAT and EU ETS experience the same direction extremal volatility. For the current trading day, λ for (CCU_n_, EUA_n_) of the corresponding is 0.1591, means the conditional probability of EUA price decreasing when CCU price falls 15.91%, which means it would occur 15.91 days among 1000 days for a current trading date, i.e., approximately 5 days per year. The conditional probability of EUA price increasing when CCU price increase is 19.99%, which means it probably occur 19.99 days among 1000 days for a current trading date, approximately 7 days per year. The empirical process is also proceeded for the 1-day lag, 2-day lag and 5-day lag. From the results shown in Table 5, we could conclude that the extremal co-movement is relevant consistent, that the same-sign-pairs are significant between the two carbon markets. Moreover, the TQCC index is stable, around 15% conditional probability for same direction extremal co-movement.

The phenomena may be explained by the mature market performance and resonable operation mechanism in CA CAT and EU ETS. Firstly, similar emisson quota supply structure influences the carbon prices operation in these two carbon markets. The EU ETS has decreasing total emission caps over its three phases. The total cap declines from 2181 million tonne allowances to 2083 million tonne allowances each year during the first phase (2005–2007) and the second phase (2008–2012) [50]. As to the third phase (2013 to 2020), the cap shrinks at 1.74% of the average total quantity of allowances issued. Similar to the EU ETS, the California carbon market set caps less than the emission level of the previous emission, specifically, 2% below in the initial phase (2012–2014) and 3% below for the second (2015–2017) and third phase (2018–2020). Secondly, various finacial products and high trading activities ensure the carbon prices stable. Both two carbon markets are most strongly influenced by coal markets, crude oil markets, gas markets and stock markets [11–15]. Thus, the two markets exist a same sign tail dependence to a certain degree.

The findings have significant guiding to the investors’ behavior. The co-movements between the two carbon markets indicate that it is unwise to consider CCU and EUA as a portfolio as the same sign pairs occur tail dependence between these two carbon assets. The tail dependence structure of CCU and EUA is significant for their same sign pairs at current day, 1 day lag, 2 day lag and 5 day lag. The result indicates that investors can predict whether EUA price has a great drop or increase on conditional of CCU’s sharp drop or increase. The empirical results draw insight of the investment strategies for the investors. As to the carbon market operation, the results show that these two carbon markets have regular tail dependence structure, illustrating that the operation of CA CAT and EU ETS are stable and order.

#### 4.2.2 Tail dependence results of EU ETS and China’s carbon pilots

In this section, we examine whether there is an extremal co-movement between EU ETS and China’s carbon markets. The estimated procedure follows Section 4.1. The empirical results displayed in Table 6 indicate that all five carbon trading pilots in China have extremal co-movement with the EU ETS. However, the co-movements between the EU ETS and China’s carbon trading pilot are different. The results show that Beijing, Shenzhen, and Hubei have a tail dependent relationship with the EU ETS on (n*n), (p*p) and (p*n) directions. Guangdong has a tail dependent relationship with the EU ETS on (n*n) and (p*n) directions. This means carbon price in Guangdong has extremal decrease only if extreme movement occurred in EU ETS. Hubei has co-movement with EU ETS on (n*n), (n*p) and (p*p) directions. Shanghai has extremal co-movement with EU ETS for all four directions.

**Table 6:** Tail dependence test result for the EU ETS and China’s carbon market.

Pair	n*n	n*p	p*p	p*n
(EUA, BJEA)	0.1789*	0.0525	0.2062*	0.2014*
	(0.0147)	0.4941	(0.0002)	(0.0182)
(EUA, GDEA)	0.4193*	0.1109	0.1491	0.2020*
	(0.0000)	(0.0896)	(0.0558)	(0.0094)
(EUA, HBEA)	0.2265*	0.1827*	0.1516*	0.0692
	(0.0000)	(0.0007)	(0.0203)	0.1907
(EUA, SHEA)	0.2165*	0.3157*	0.2913*	0.3990*
	(0.0158)	(0.0011)	(0.0000)	(0.0018)
(EUA, SZEA)	0.1816*	0.0261	0.2915*	0.2393*
	(0.0002)	(0.6603)	(0.0000)	(0.0000)

* means significant at 0.05.

The reason for existing tail dependence may be due to the similar mechanism design. As the EU ETS is the most effective and influenced carbon market in the world, China’s carbon trading pilots follow it on designing the trading scheme. From the demand aspect, the regulated companies are emission intensive companies in EU ETS and China’s carbon market. As Table 7 shown, both EU ETS and China’s carbon markets cover the following industries including power and heat generation, oil refineries, steel and iron and some other energy-intensive sectors. From the quota supply aspect, similar to the EU ETS, the carbon trading pilots in China are also mandatory markets. It means the enterprises emitting over a certain amount have to attend to carbon market to surrender allowances for reported emissions. In addition, allowances allocation is mainly based on the former emissions (grandfathering method). Most allowances are distributed freely especially in the first phase. As summarized in Table 7, 95% allowances are distributed freely in the EU ETS for its initial phase, and China’s carbon trading pilots allocate their permits freely ranging from 90% to 100%. Therefore, carbon prices are under similar price operation mechanism.

**Table 7:** Covered sectors in the EU ETS and China’s carbon trading pilots.

Exchange	EU ETS (the first phase)	China				
		Beijing	Guangdong	Hubei	Shanghai	Shenzhen
Covered sectors	Power, petrochemical, iron and steel, building materials, paper, aviation	Industrial sectors and service sectors	Power, iron and steel, cement, petrochemicals, aviation, paper and cement	Power, iron and steel, nonferrous metals, cement, building materials, pulp and paper	Industrial sectors, building sectors	Power, water supply, manufacturing, building
Free distribution	95%	95%	97%	90%	100%	95%

Source: individual carbon trading market website

The extremal relationship is different and in chaos between China’s carbon trading pilots and the EU ETS. Firstly, different from EU ETS, China’s carbon markets are under controlled by the government. National Development and Reform Commission (NDRC) makes the carbon trading principles and regulate most covered enterprises in China. The non-marketization makes the tradeable asset with low liquidity and trading volume, as ETS is a fundamentally market-based instrument [51]. Secondly, the inactivity of carbon markets in China influences the tail dependence results. China’s carbon markets are under low turnover and more non-trading days. For instance, the turnover of Beijing ETS and Hubei ETS is higher than other carbon markets with just 5.16% and 3.18% respectively. As to the non-trading days, Guangdong ETS and Beijing ETS have 39 and 40 non-trading days among 227 trading days in 2016 respectively [52]. Thirdly, carbon prices are closely related to energy prices. However, the influences of global energy market fluctuation on China’s carbon markets are uncertain. As the energy prices in China are not market driven. For instance, China’s electricity market price is subject to the NDRC and adjust the electricity price not regularly. China’s oil market price adjustment is only changed when international oil prices change and reach a certain level and last for a certain period. In summary, carbon market in China is not mature and it is still in trial phase. The ETS regulations lack a strong legal basis in China. In general, poor enforcement of environmental regulations and the scarcity of publicly data on CO_2_ emissions are the problems for China's implementation of ETS.

#### 4.2.3 Tail dependence results for China’s carbon markets

The tail dependence results among China’s carbon markets are shown in Table 8. From the empirical results, we could conclude that carbon markets in China exist tail dependent tendency. Seven of ten combinations have significant tail dependence for two pairs. Among them, the opposite-sign-pairs of (SZEA, HBEA) and (HBEA, GZEA) respectively are significant. The λ of (SZEA_n_, HBEA_p_) is 0.3107, which means the conditional probability of Hubei increases when SZEA drops is 0.31066. In contrast, the conditional probability of Hubei drops when SZEA raises is 0.3768. The interpretation of (HBEA, GZEA) is similar. Given the opposite-sign-pairs being significant, investors could use this kind of pairs to avoid risk. The same-sign-pairs of (HBEA, BJEA) and (HBEA, SHEA) have tail dependence structure. This means Beijing carbon market and Shanghai carbon market move up or down extremally with Hubei carbon market in the same direction. (SZEA_n_, BJEA_p_) and (SZEA_p_, BJEA_p_) are significantly tail dependent. (SZEA_p_, SHEA_p_) and (SZEA_p_, BJEA_n_) are significant among all pairs between these two markets. Comparing the pairs between Shanghai and Guangzhou, (SHEA_n_, GZEA_n_) and (SHEA_p_, GZEA_n_) are significant, which means carbon price in Guangzhou carbon market has extremal decrease only if there is extremal movement in Shanghai carbon market. (SZEA_p_, GZEA_p_) and (BJEA_p_, GZEA_p_) are significant among all pairs between the related two carbon markers respectively. Carbon price raises in Guangzhou has a high tendency on condition of carbon prices increase in SZEE and Beijing. Carbon prices in Guangzhou and Shanghai have high conditional probability movement in three directions, including (n*n), (n*p) and (p*n).

**Table 8:** Tail dependence test result: Among China’s carbon markets.

Pairs	n*n	n*p	p*p	p*n
(SZEA, HBEA)	0.1049	0.3107*	0.0247	0.3768*
	(0.0689)	(0.0000)	(0.7894)	(0.0000)
(SZEA, BJEA)	0.0384	0.1325*	0.2550*	0.0555
	(0.5212)	(0.0349)	(0.0062)	(0.4777)
(SZEA, SHEA)	0.1034	0.0392	0.3687*	0.1829*
	(0.1946)	(0.5535)	(0.0001)	(0.0035)
(SZEA, GDEA)	0.0951	0.0804	0.1383*	0.2127
	(0.1501)	(0.3005)	(0.0427)	(0.0838)
(HBEA, BJEA)	0.1885*	0.1325	0.3156*	0.0510
	(0.0453)	(0.0610)	(0.0004)	(0.6111)
(HBEA, SHEA)	0.1329*	0.1046	0.5355*	0.1867
	(0.0163)	(0.3408)	(0.0000)	(0.1816)
(HBEA, GDEA)	0.1177	0.1976*	0.0587	0.2826*
	(0.0793)	(0.0109)	(0.3713)	(0.0006)
(BJEA, SHEA)	0.5225*	0.5892*	0.0421	0.7334*
	(0.0002)	(0.0006)	(0.5453)	(0.0001)
(BJEA, GDEA)	0.1309	0.1120	0.1931*	0.1138
	(0.3303)	(0.0696)	(0.0094)	(0.0864)
(SHEA, GDEA)	0.2730*	0.1006	0.1396	0.4963*
	(0.0271)	(0.2451)	(0.2623)	(0.0000)

* means significant at 0.05.

From the estimation results, we could also find that the extremal relationship directions are various. The reasons for existing co-movements may be inferred as follows. Firstly, government influences carbon markets in China at a great extent. On the one hand, China’s carbon markets design and development are led by NDRC. As they are still at the trial stage, marketization in carbon markets is not implemented completely. China’s carbon markets are highly sensitive to policy releasement. For instance, China’s government not only controls price but also encourage financial institutes to give financial support to the energy conservation and environmental protection equipment improvement. On the other hand, carbon markets participants in China are mainly pollution intensive enterprises, such as power corporates, coal companies and iron and steel firms. These enterprises are mainly state-owned enterprises, which are subject to the government regulations. Secondly, compliance events of China’s carbon markets are around June and July in each year. Emissions dealings in China are not continuous, which makes market participants are difficult to predict carbon prices and make asset portfolio management. Many investors hold a conservative attitude on this kind of emerging markets. As carbon quotas are closely related to energy-intensive companies including production procedure and consumption procedure, companies tend to hold excess emission allowances but not sell out in case of later demand. Since the carbon allowances in China cannot be saved and used in the further compliance period, trading volume significantly spikes near the compliance time. During the performance periods, enterprises emitting over than the limit would buy quotas in carbon market to avoid punishment, while enterprises who have redundant allowances would sell excess quotas. This would bring about price volatility to all carbon markets. Thirdly, quota allocation method is similar in all China’s carbon markets. Quota allocation gives priority to free quota allocation, accounted for 90–100%.

The results indicate that carbon markets in China are in chaos without having formed routine market laws. Carbon pilots in China are with high risks since the cost of buying quota represents a tiny proportion of the total production costs for the pollution-intensive producers. Pursuing higher economic interests, pollution-intensive producers probably pay little attention to fine when their emissions exceed the limit. The prices of carbon quota are not attractive. Market participants prefer to reserve surplus quota in case of quota shortage later. The directions of extremal co-movement are various since the implementing policies are inconsistent in these five carbon markets. Carbon trading pilots are not only controlled by NDRC but also are regulated by the local government. The specific operating systems are designed by the local DRC. For example, Hubei carbon trading pilot encourages investors and regulated sectors to trade actively. The carbon allowances distributions are considered as a local rapid economic growth expectation, industrial development and existing covered enterprises’ emission data. For the Shenzhen carbon trading pilot, it is the only market that allocates all three years’ allowances at once and allows the market participants used the redundant from former compliance period [53].

### 4.3 Illustration of individual pilot

In general, extremal co-movements of China’s carbon markets are not obvious. This is mainly due to the gap between allocated allowances and actual emissions of regulated enterprises. The loose carbon quota policy is launched considered expectations of rapid economic development, non-transparent production information, and covert emission data. We note that Hubei carbon trading pilot is an expectation. Hubei carbon trading pilot has tail dependence with other four carbon markets at a certain degree. Beijing carbon pilot and Shanghai carbon pilot have the same-position tail dependence with Hubei carbon pilot respectively. Shenzhen carbon pilot and Guangdong carbon pilot have extremal co-movements with Hubei carbon pilot respectively.

Comparing with other carbon pilots, Hubei carbon trading pilot remained more liquid with an average daily volume of 47,995 tons in the sample period. Hubei NRC stipulate that the surplus allowances would not be used in the next compliance period. The emission data collection process in Hubei is efficient, and the control in allowances is strict with dynamic adjustment. The scheme allows the program administrator to take back the regulated enterprises’ surplus allowances led by a drop in industrial production. It also requires the administrator to cancel the reserved allowances for new entrants that would not be distributed until the compliance date. As a result, the quota quantities would be reduced in the following compliance year.

### 4.4 Risk transmit mechanisms among carbon markets

The existing carbon markets transmit the risks by the following patterns:

Firstly, carbon markets risk transmission takes energy prices as a medium. Carbon markets aim at controlling GHG emissions and mitigating climate change. Thus, energy-intensive enterprises and pollution-intensive enterprises are mainly regulated by carbon markets. Carbon markets are affected by various variables, such as energy prices, stock prices, regulation policies and so on [5–14]. The existing studies verify that the carbon prices are related to energy prices, such as power prices, coal prices, gas prices and renewable stock prices. Carbon prices in CA CAT, EU ETS and China are influenced by energy prices volatility. However, energy prices in China are not only determined by supply and demand, but also by government intervention. Thus, the risk transmission between CA CAT and EU ETS are bilateral with same sign pair extremal co-movements. The risk transmission between China’s carbon markets and EU ETS is irregular.

Secondly, the rationale behind the risk transmission is that the EU ETS, as the biggest carbon market, has a great impact on carbon markets in other regions. EU ETS is the oldest carbon markets in the world, established in 2005 [4]. Both CA CAT and China’s carbon markets refer the experience of EU ETS on operating carbon markets. Thus, analyzing the tail structure results of carbon markets in U.S., Europe and China is crucially figuring out the operation mechanism problems of China’s carbon markets.

Thirdly, although there are currently no direct bilateral links between carbon markets, the existing carbon markets are linking to the Clean Development Mechanism (CDM). Linking between carbon markets are occurred indirectly. CDM allows emitters to meet the emission reduction goal by offsets the emission reduction behavior in other regions [54,55]. Thus, the CDM influences the carbon prices in different regions and leads to indirect linking between the ETSs.

## 5. Conclusion and implication

To sum up, the extremal co-movements exist between carbon markets located in different countries and regions. The findings provide some valuable insights for the investors in risk management, and policy makers. The tail dependence result of the CA CAT and the EU ETS indicates that it is unwise to take asset portfolio including CCU and EUA. The results for the EU ETS and China’s carbon markets show that the dependence is not regular, mainly due to the development characteristics and disorder conduction of China’s carbon markets. ETS regulations lack a strong legal principle. Poor implementation of environmental regulations and scarcity of publicly available data on carbon emissions are the crucial problems for executing ETS in China. There is seldom probability of benefiting or avoiding risks by holding theses portfolios. As to the extremal co-movements in China’s carbon markets, investors can trade (SZEA, HBEA), (HBEA, GZEA) as investment portfolios respectively to hedge risks. The traders can also raise their profits and decrease their investment risks by holding the portfolios of (SZEA, GZEA), (HBEA, BJEA), (HBEA, SHEA) and (BJEA, GZEA).

From the result, we can conclude that China’s ETS is not effective for its over-allocated quota distribution [56]. As a financial instrument, ETS in China only exists the spot trading but no futures or options. It affects the validity of allowances and the diversity of financial products. China’s carbon market is not a mature, functional capital market yet. Imperfect financial attributes make unstable carbon prices, which affects market liquidity. Intensive pollution enterprises have a basic understanding of carbon market mechanism and carbon asset management, but weak carbon trading willingness. Enterprises take part in carbon market trading just for compliance. Most regulated enterprises are state-own companies, who pay little attention to gain benefit from emissions trading and participate in carbon market trading for compliance and fulfilling social responsibility [57]. Moreover, transactions are intervened by government, as China’s carbon markets have sharp governmental characteristics. Emissions trading is mainly driven by Chinese government executive order, i.e., the first dealing in Shanghai carbon markets is conducted by the local government consultation. The non-market pressure will cause abnormal price fluctuation.

Based on the empirical results and the individual analysis of Hubei carbon trading pilot, we will give some implications for China’s ETS as follows especially for its sustainable aspect. Firstly, carbon assets could be considered when investors allocate their assets. Carbon assets trading not only benefit investors but also have a positive impact on environmental improvement. Secondly, as mentioned before, the management of ETS in China is disorder. The existing study has recognized the overall cap stringency made by political and institutional. China has tried to adapt the CO_2_ emission trading scheme to China’s actual situation by crafting deigns deviating from international approaches. The adaption represented deft tailoring of a carbon market to the local context. The design is made according to the different characteristics of the respective districts. The government or the carbon markets coordinators should improve system design, information platform, allowance allocation. Thirdly, the regulated enterprises in China should increase their environmental protection awareness, pay more attention to clean production and participate in the carbon market actively. Only in this way, the carbon market in China will operate in an order and healthy pattern and achieve its emission reduction goal.
